# Pan-Cancer Analysis of GPR141: Unveiling its prognostic significance, immune microenvironment interactions, and therapeutic potential

**DOI:** 10.7150/jca.118222

**Published:** 2025-08-11

**Authors:** Jingyue Sun, Sha Qin, Zisheng Li, Xin Peng, Desheng Xiao, Yongguang Tao, Bin Xie

**Affiliations:** 1 Key Laboratory of Carcinogenesis and Cancer Invasion, Ministry of Education; Department of Pathology, Xiangya Hospital, Central South University, Hunan, 410078, China.; 2 NHC Key Laboratory of Carcinogenesis of Ministry of Health (Central South University), Cancer Research Institute; School of Basic Medicine, Central South University, Changsha, Hunan, 410078, China.; 3 Department of Pathology, Xiangya Hospital, Central South University, Changsha, Hunan, 410008, China.

**Keywords:** GPR141, prognosis, pan-cancer, tumor biomarkers

## Abstract

**Background:** GPCRs play an important role in the development of cancer. However, as a member of the G protein-coupled receptor family, the function of GPR141 is still unclear, and its function in pan-cancer is even less known.

**Methods:** In this study, a series of bioinformatics methods were used to explore the potential carcinogenic effects of GPR141, including analysis of GPR141 expression in different tumors, related prognosis, mutations, gene correlation analysis, gene enrichment analysis, immune cell infiltration and other factors. All results and data are available from TIMER, GEPIA2.0, TISIDB, cBioportal and other data portals. In addition, we collected lung adenocarcinoma samples, hepatocellular carcinoma and adjacent tissues for immunohistochemical analysis. And we stably knocked down GPR141 in lung adenocarcinoma cell lines A549 and H1975, and the changes of cell proliferation, migration and invasion were detected by CCK8, Transwell migration and invasion experiments.

**Results:** GPR141 is differentially expressed in a variety of cancers. GPR141 is closely related to the prognosis, genetic changes and immune infiltrating cells of tumor patients. Gene enrichment analysis showed that GPR141 was mainly involved in immune-related pathways in pan-cancer. In pan-cancer, the expression levels of PTPRC, TLRB, PLEK, NCKAP1L, PGS18 and CLEC12A were positively correlated with GPR141. Immunohistochemical results showed that the expression of GPR141 in lung adenocarcinoma and hepatocellular carcinoma was higher than that in adjacent tissues. After knocking down GPR141 in A549 and H1975, we found that the proliferation, migration and invasion of lung adenocarcinoma cells decreased after knocking down GPR141.

**Conclusion:** GPR141 may be a new prognostic marker and therapeutic target for human tumors, providing a theoretical basis for the development of more effective and targeted clinical treatment of cancer.

## Introduction

Cancer has become a significant threat to human health. Scientific investigations have identified numerous etiological factors contributing to oncogenesis. With the progressive evolution of medical science, therapeutic modalities for malignant neoplasms have substantially expanded beyond conventional approaches including surgical intervention, cytotoxic chemotherapy, and radiation therapy. Contemporary oncological research has witnessed remarkable advancements in novel treatment strategies, particularly in the development of molecularly targeted therapies, photodynamic interventions, and photothermal ablation techniques, which are increasingly being implemented in clinical practice 1-8. In parallel with other therapeutic advancements, immunotherapeutic approaches have emerged as a pivotal strategy in oncology. The clinical application of immune checkpoint blockade, particularly through inhibitors targeting programmed cell death protein 1 (PD-1), its ligand (PD-L1), cytotoxic T-lymphocyte-associated protein 4 (CTLA-4), and lymphocyte-activation gene 3 (LAG-3), has demonstrated significant clinical benefits across multiple malignancies. These immunomodulatory agents have revolutionized cancer treatment paradigms, showing unprecedented response rates and durable clinical outcomes in various tumor types 9-15. But even so, the prognosis of cancer is still poor. Therefore, we need to continue to study and analyze more effective molecules to guide our treatment of tumors.

As the most extensive group of membrane-bound proteins encoded in the human genome, G protein-coupled receptors (GPCRs) constitute the largest family of cell surface signaling molecules. These transmembrane receptors are systematically categorized into five distinct classes based on their structural and functional characteristics: class A (rhodopsin-like receptors), class B1 (secretin receptor family), class B2 (adhesion GPCRs), class C (metabotropic glutamate/calcium-sensing receptors), and class F (frizzled/smoothened and taste2 receptors) 16-22. These membrane-bound signaling molecules exhibit ubiquitous expression patterns throughout major organ systems, where they serve as crucial modulators of developmental processes and homeostatic maintenance. Their functional significance is particularly evident in several vital anatomical regions: the neural circuitry of the brain and spinal cord, hematopoietic and lymphoid tissues, the circulatory network, and ocular photoreceptive structures. Through their diverse localization, these receptors orchestrate a wide spectrum of biological processes essential for organismal function and survival 23-32.

As an orphan receptor belonging to the class A rhodopsin family, GPR141 exhibits unique structural characteristics that distinguish it from typical rhodopsin-like GPCRs. Notably, in its third transmembrane helix, this receptor possesses a distinctive TRY amino acid sequence at the position where most rhodopsin-family receptors maintain a conserved DRY motif. Furthermore, structural analysis reveals the absence of another characteristic sequence pattern - the NSxxNPxxY motif - within its seventh transmembrane domain, which represents a hallmark feature of conventional rhodopsin-like G protein-coupled receptors. These structural deviations suggest potential functional and signaling pathway differences compared to canonical class A GPCRs 33-37. The physiological functions and endogenous ligands for GPR141 remain unclear. However, study has shown that GPR141 can affect the development of breast cancer through mTOR / p53, and GPR141 exists as an immunosuppressive factor in multiple sclerosis 33, 38. Emerging research findings suggest that GPR141 may represent a promising therapeutic target for oncological interventions. Nevertheless, the comprehensive functional mechanisms and pathological implications of this receptor across various cancer types have not been fully elucidated, warranting further systematic investigation to clarify its role in tumor biology and potential clinical applications.

This investigation employed comprehensive bioinformatics approaches to systematically analyze GPR141 across multiple cancer types, focusing on its expression patterns, prognostic significance, genomic variations, and immunological associations. Our multi-dimensional examination uncovered several critical aspects: the prognostic utility of GPR141 in diverse malignancies, its potential involvement in previously uncharacterized cancer types, the molecular pathways through which it contributes to oncogenesis, and its impact on tumor immunology. Furthermore, functional enrichment analyses provided insights into the biological processes and signaling networks associated with GPR141 in cancer development and progression.

## Materials and Methods

### Gene expression analysis

Differential expression patterns of GPR141 between malignant and adjacent normal tissues were evaluated across multiple cancer types using the differential expression module within TIMER2 (http://timer.cistrome.org/), a comprehensive platform for tumor immunology research. To assess the association between GPR141 transcriptional levels and disease progression, we employed GEPIA2.0 (http://gepia2.cancer-pku.cn/) to examine its correlation with pathological staging parameters across all cancer types available in The Cancer Genome Atlas (TCGA) database.

### Survival prognosis analysis

Patient survival analysis was conducted through survival module of GEPIA2.0 (http://gepia2.cancer-pku.cn/), which generated Kaplan-Meier curves to evaluate both overall and disease-free survival outcomes. Additionally, a comprehensive significance map was constructed to visualize the prognostic relevance of GPR141 expression across all TCGA tumor types, providing a systematic overview of its clinical implications in various malignancies.

### Immunogenomic analysis

To comprehensively evaluate the association between GPR141 expression patterns and tumor immune microenvironment characteristics, we employed multiple computational algorithms including EPIC, MCPCOUNTER, QUANTISEQ, TIDE, TIMER, and XCELL through the TIMER platform. These bioinformatics tools were utilized to assess immune cell infiltration levels across all cancer types available in the TCGA database. Additionally, systematic analysis was conducted using the TISIDB repository to examine potential relationships between GPR141 expression profiles and various immune-related components, encompassing immunomodulatory molecules, major histocompatibility complex (MHC) proteins, chemokine ligands, and their corresponding receptors in a pan-cancer context.

### Gene alteration analysis

Genomic alteration analysis of GPR141 was performed using the cBioPortal platform (https://www.cbioportal.org/) through interrogation of the TCGA PanCancer Atlas dataset. The investigation of mutational patterns and variant localization was conducted utilizing three analytical components: the Oncoprint visualization tool, tumor-type specific genomic summaries, and the mutation profile module, which collectively enabled comprehensive characterization of genetic modifications across diverse malignancies.

### GPR141-related gene enrichment analysis

The protein-protein interaction network for GPR141 in Homo sapiens was constructed using STRING (version 11.0b; https://string-db.org/) with specific configuration parameters: co-expression as the primary interaction source, evidence-based edge representation, a maximum of 50 interacting partners, and a minimum interaction confidence threshold set at 0.150.

Through the gene similarity module of GEPIA2.0, we identified the top 100 genes exhibiting the highest co-expression patterns with GPR141 across TCGA datasets. These candidate genes were subsequently subjected to Gene Ontology analysis using the clusterProfiler package (v3.13) in R to elucidate potential biological pathways. Furthermore, we conducted bivariate correlation assessments between GPR141 and its co-expressed genes using GEPIA2's correlation analysis component.

### Cell culture and plasmids

A549 cells were grown in DMEM/F12(Gibco), H1975, H23, H358, H1299 cells were grown in 1640 (Gibco). BEAS-2B cells were grown in DMEM (Gibco). Culture medium was supplemented with 10% bovine calf serum (B7446, Sigma-Aldrich) and 1% penicillin/streptomycin/gentamicin.

The sh-GPR141 targeting regions, ACCAGTTCTTTAGGATCTATT (sh-GPR141 #1) and GTTCCTATTCTATGTGGTGAT (sh-GPR141 #2) were inserted into pLVX-shRNA1.

### CCK8 viability assay

The cells were cultured in 96-well plates and treated with the indicated reagents. After treatment, 10 µL of Cell Counting Kit-8 (CCK-8) reagent (Bimake) was added to each well, followed by incubation for 2 h. After incubation, the absorbance at 450 nm was measured using a microplate reader (BioTek).

### Transwell migration assay

A549 and H1975 cells were resuspended in serum-free medium and seeded into the upper chamber of a 24-well Transwell system (Corning, 8-μm pore size) at a density of 5 × 10⁴ cells/200 μL. The lower chamber was filled with medium containing 10% serum as a chemoattractant. After 24 h, cells on both sides of the membrane were fixed with methanol and stained with crystal violet. Non-migrated cells in the upper chamber were gently removed using a wet cotton swab. Five randomly selected fields per well were photographed and analyzed under a microscope.

### Transwell invasion assay

Matrigel: serum-free medium is 1:9, and 60 μL of the mixture was added to each chamber. A549 and H1975 cells were resuspended in serum-free medium and seeded into the upper chamber of a 24-well Transwell system (Corning, 8-μm pore size) at a density of 1 × 10^5^ cells/200 μL. The lower chamber was filled with medium containing 10% serum as a chemoattractant. After 48 h, cells on both sides of the membrane were fixed with methanol and stained with crystal violet. Non-migrated cells in the upper chamber were gently removed using a wet cotton swab. Five randomly selected fields per well were photographed and analyzed under a microscope.

### Western blot

Cells were washed three times with 1x PBS. The cells were resuspended using IP lysis buffer with protease inhibitor, and then the cells were lysed on ice. Total protein lysates were resolved by SDS-polyacrylamide gel electrophoresis (SDS-PAGE) and electrophoretically transferred to a polyvinylidene fluoride (PVDF) membrane. The applied antibodies were as follows: anti-GPR141(orb-183868) was purchased from Biorbyt; anti-β-Actin(A1978) was purchased from sigma; Normal Rabbit IgG (7073s) and Normal Mouse IgG(7076s) were purchased from Cell Signaling Technology.

### Histology and immunohistochemistry

We collected 10 cases of lung adenocarcinoma, 10 cases of hepatocellular carcinoma and their corresponding paracancerous tissues from Xiangya Hospital of Central South University. This study was approved by the Institutional Review Board of Xiangya School of Basic Medical Sciences, Central South University. To assess the protein expression level of GPR141 gene in tumor and corresponding paracancerous tissues, we conducted an immunohistochemical analysis.

Following routine deparaffinization and hydration, tissue sections underwent treatment with 3% hydrogen peroxide to block endogenous peroxidase activity. Antigen retrieval was subsequently performed by heating the sections in sodium citrate buffer. Primary antibody incubation utilized anti-GPR141 (diluted 1:100) at 4°C overnight. Sections were then incubated with the appropriate secondary antibody at room temperature for 30 minutes. Two independent pathologists from Xiangya Hospital, Central South University, China, performed blinded evaluation of all stained slides to ensure scoring consistency. GPR141 expression was assessed using a dual-parameter scoring system. Staining intensity was categorized as: 0 (negative), 1 (buff), 2 (pale brown), or 3 (tan). The percentage of positive tumor cells was scored as: 0 (negative), 1+ (<10%), 2+ (11-50%), 3+ (51-75%), or 4+ (>75%). The final immunohistochemical score was determined by multiplying the intensity score by the positivity percentage score.

## Results

### GPR141 expression in human pan-cancer

**Figure 1:**
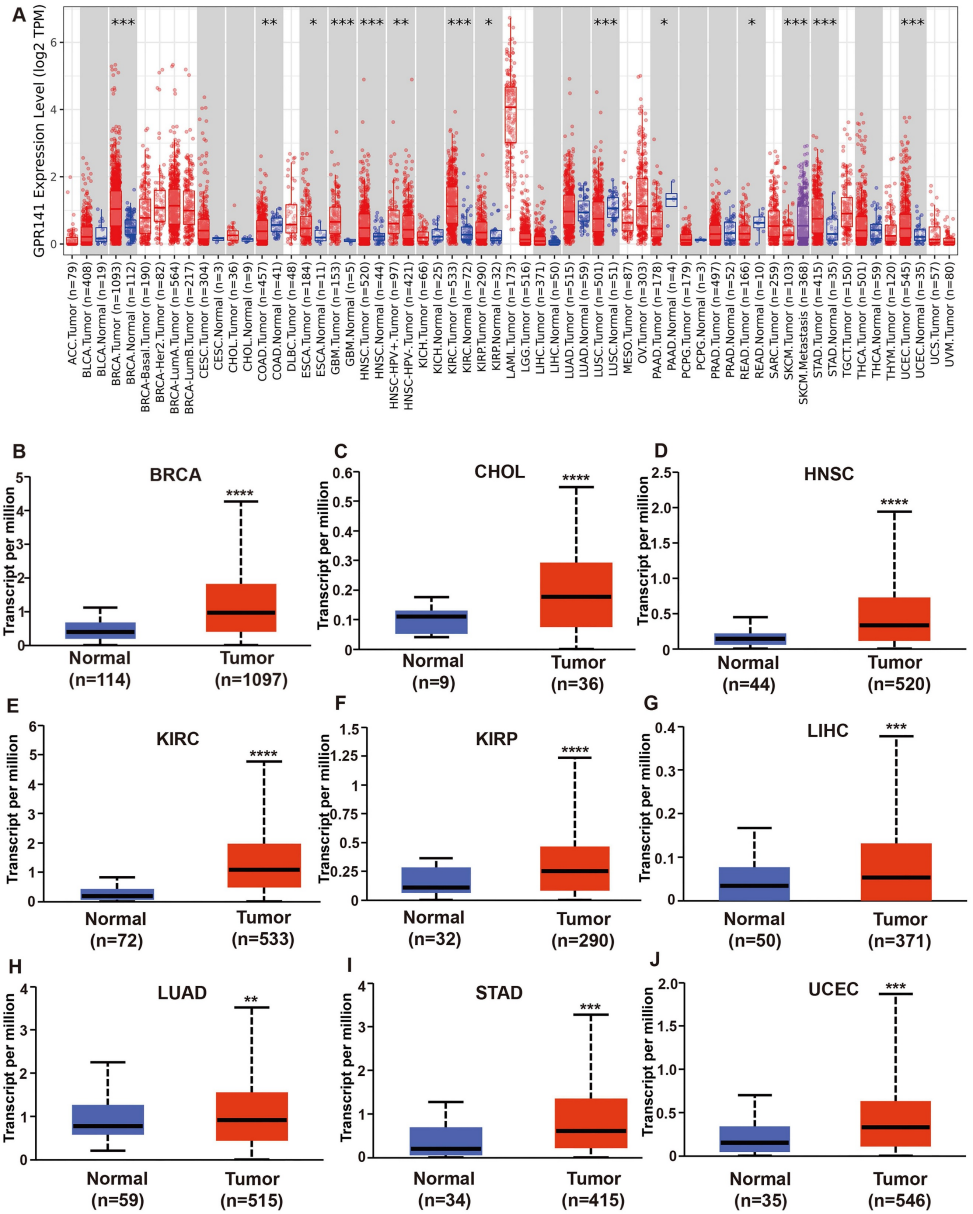
** The mRNA expression levels of GPR141 in human pan-cancer.** (A) The expression status of GPR141 in different tumor types was visualized by TIMER2.0 *p < 0.05; **p < 0.01; ***p < 0.001. (B-J) GPR141 mRNA expression level in normal tissues and cancers from UALCAN.

We analyzed the mRNA expression levels of GPR141 in pan-cancer by TIMER (Figure 1A), and the list of abbreviations for all cancers analyzed were included in Supplementary Table 1. The analysis of GPR141 mRNA expression between paracancerous tissues and cancers revealed that GPR141 expressed significantly higher in BRCA (Breast invasive carcinoma), ESCA (Esophageal carcinoma), GBM (Glioblastoma multiforme), HNSC (Head and Neck squamous cell carcinoma), KIRC (Kidney renal clear cell carcinoma), KIRP (Kidney renal papillary cell carcinoma), STAD (Stomach adenocarcinoma), UCEC (Uterine Corpus Endometrial Carcinoma), and significantly lower in COAD (Colon adenocarcinoma), LUSC (Lung squamous cell carcinoma), PAAD (Pancreatic adenocarcinoma), READ (Rectum adenocarcinoma). There was no obvious difference shown in BLCA (Bladder Urothelial Carcinoma), CESC (Cervical squamous cell carcinoma and endocervical adenocarcinoma), CHOL (Cholangiocarcinoma), KICH (Kidney Chromophobe), LIHC (Liver hepatocellular carcinoma), LUAD (Lung adenocarcinoma), PCPG (Pheochromocytoma and Paraganglioma), PRAD (Prostate adenocarcinoma), THCA (Thyroid carcinoma). ACC (Adrenocortical carcinoma), DLBC (Lymphoid Neoplasm Diffuse Large B-cell Lymphoma), LAML (Acute Myeloid Leukemia), LGG (Brain Lower Grade Glioma), MESO (Mesothelioma), OV (Ovarian serous cystadenocarcinoma), SARC (Sarcoma), SKCM (Skin Cutaneous Melanoma), TGCT (Testicular Germ Cell Tumors), THYM (Thymoma), UCS (Uterine Carcinosarcoma), UVM (Uveal Melanoma) were unable to be analyzed due to the lack of sufficient paracancerous samples. We also found GPR141 expressed in HNSC-HPV (+) higher than GPR141 expressed in HNSC-HPV (-). We also found GPR141 expressed SKCM metastasis higher than that in SKCM.

To further analyze, a comparative analysis of GPR141 expression in tissue samples was conducted via UCLAN. The results revealed that the expression of GPR141 was significantly elevated in several malignant tumors, including BRCA, CHOL, HNSC, KIRC, KIRP, LIHC, LUAD, STAD, UCEC, when compared with adjacent normal tissues (Figure 1B-J). Additionally, we observed that GPR141 expression was significantly higher in metastatic tumors than in primary tumors in SKCM (Supplementary Figure 1A). In LGG, Grade 3 tissues exhibited elevated GPR141 levels compared to Grade 2 tissues (Supplementary Figure 1B). Subsequent investigation using GEPIA2.0 demonstrated significant associations between GPR141 transcript levels and pathological staging in BLCA, COAD, KICH, SKCM and STAD (Supplementary Figure 1C-G).

### GPR141 expression in different molecular subtypes and immune subtypes of pan-cancer

**Figure 2:**
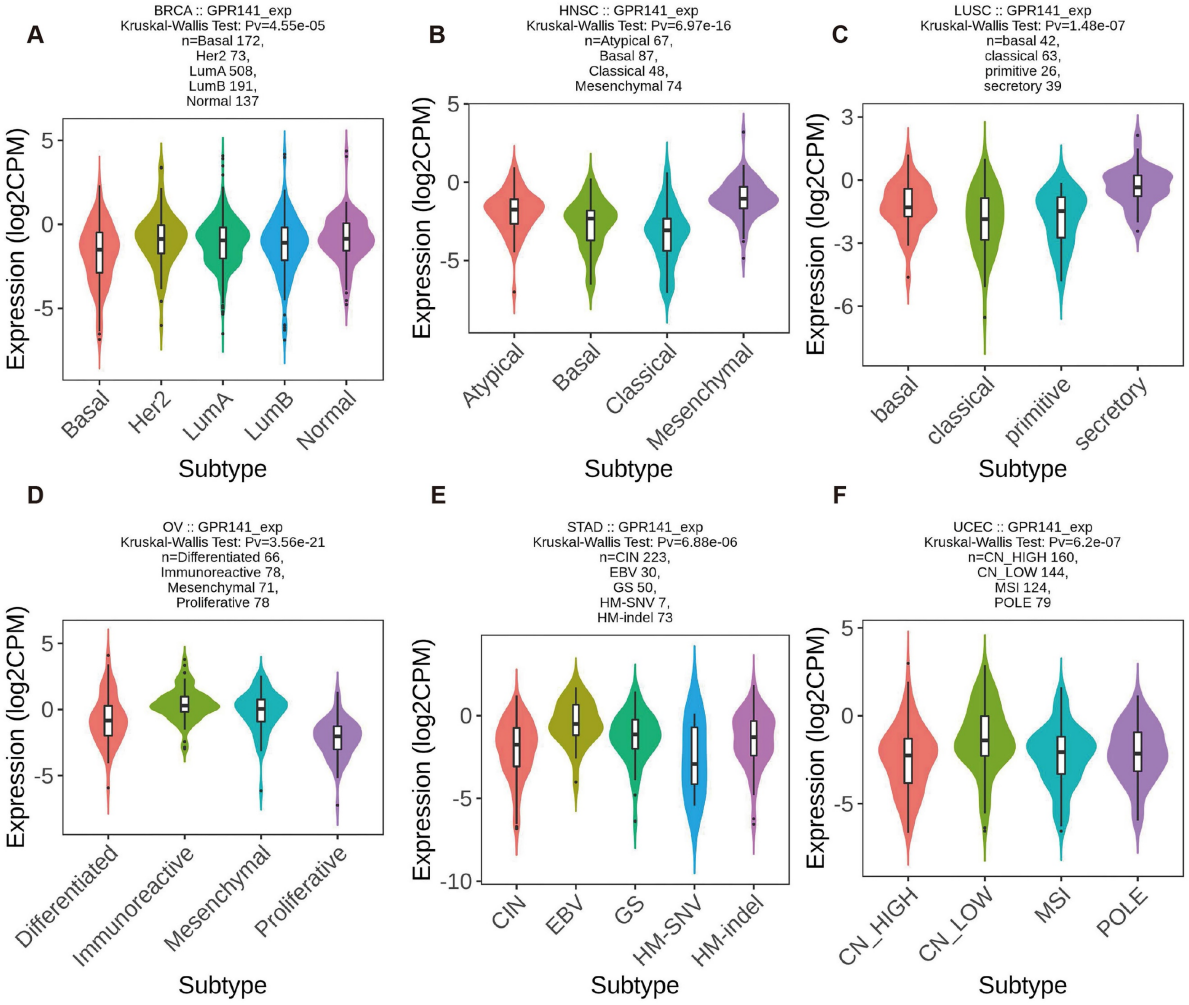
** The correlation between GPR141 expression and different molecular subtypes of human pan-cancer.** (A) BRCA. (B)HNSC. (C)LUSC. (D)OV. (E)STAD. (F)UCEC. Correlation analysis between GPR141 expression and molecular subtypes performed via TISIDB database.

**Figure 3:**
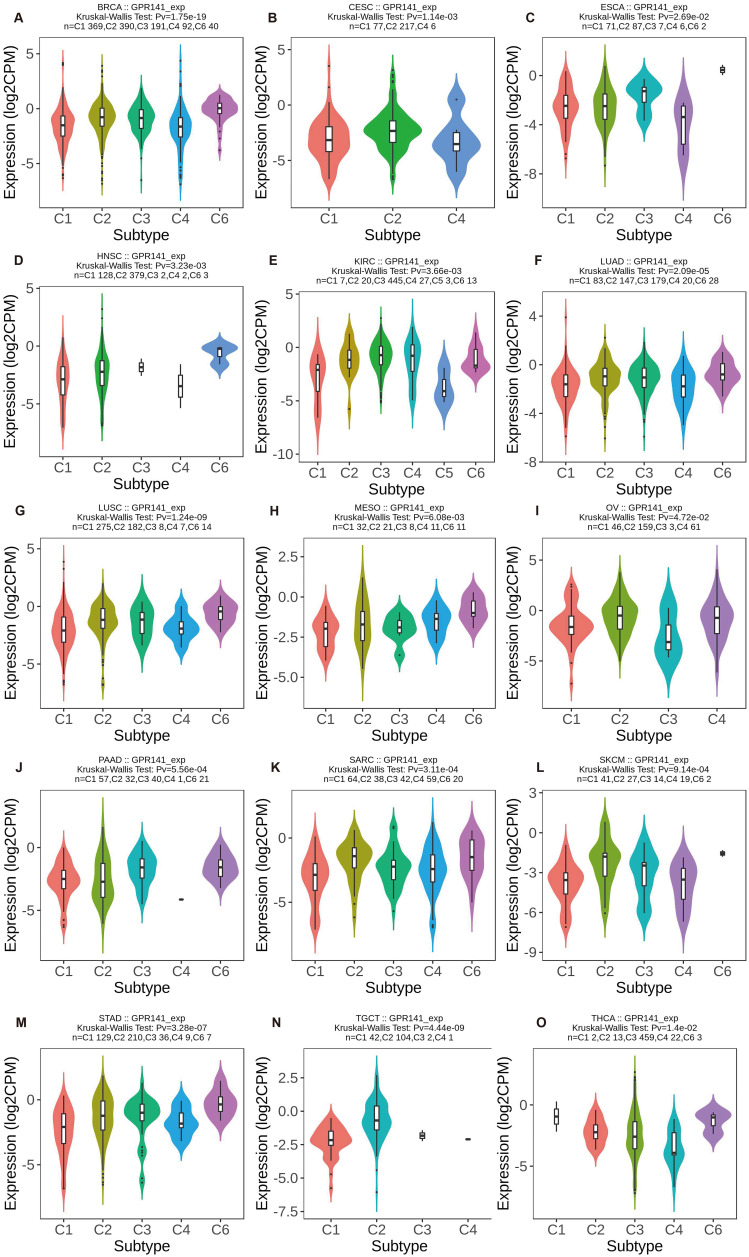
** The correlation between GPR141 expression and different immune subtypes of human pan-cancer.** (A)BRCA. (B)CESC. (C)ESCA. (D)HNSC. (E)KIRC. (F)LUAD. (G)LUSC. (H)MESO. (I)OV. (J)PAAD. (H)SARC. (L)SKCM. (M)STAD. (N)TGCT. (O)THCA. Correlation analysis between GPR141 expression and **immune** subtypes performed via TISIDB database.

We investigated GPR141 expression patterns across molecular and immune subtypes in pan-cancer using TISIDB database data. Significant differential expression of GPR141 among molecular subtypes was identified in six malignancies. GPR141 levels were highest in the Her2 subtype of BRCA (5 subtypes), Mesenchymal subtype of HNSC (4 subtypes), Secretory subtype of LUSC (4 subtypes), Immunoreactive subtype of OV (4 subtypes), EBV subtype of STAD (5 subtypes), and CN_Low subtype of UCEC (4 subtypes) (Figure 2A-F). Additionally, significant associations between GPR141 expression and immune subtypes were observed in 15 cancers. These immune categories are classified as: C1 (wound healing), C2 (IFN-gamma dominant), C3 (inflammatory), C4 (lymphocyte depleted), C5 (immunologically quiet), and C6 (TGF-b dominant). Affected cancers included BRCA, CESC, ESCA, HNSC, KIRC, LUAD, LUSC, MESO, OV, PAAD, SARC, SKCM, STAD, TGCT, and THCA (Figure 3A-O).

### The association between GPR141 expression and prognosis of human pan-cancer

**Figure 4:**
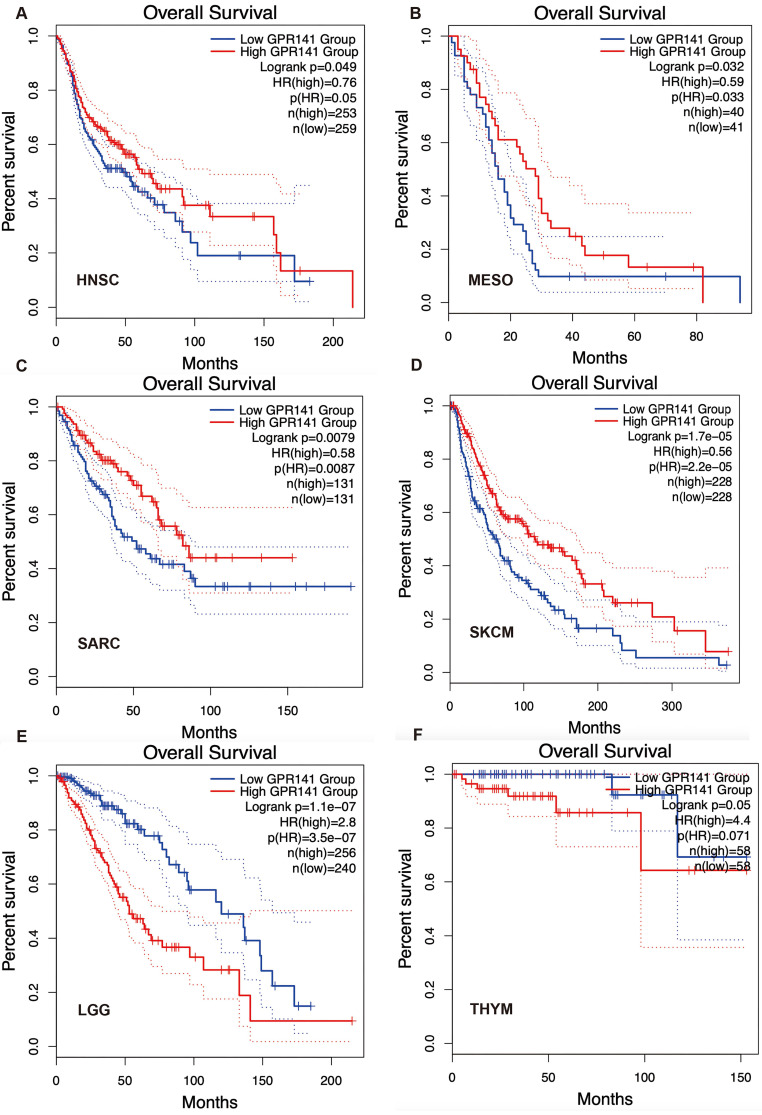
** Correlation between GPR141 expression and overall survival in human pan-cancer.** Kaplan-Meier curves for patient's overall survival classified by different expression level of GPR141 in HNSC(A), MESO(B), SARC(C), SKCM(D), LGG(E) and THYM(F).

To explore the potential prognosis value of GPR141, we investigated the correlation between GPR141 expression and prognosis of the patients with different cancers by using GEPIA2.0. The results showed that higher GPR141 expression was associated with longer OS in cases of HNSC, MESO, SARC, SKCM (Figure 4A-D). Survival analysis revealed a significant inverse correlation between GPR141 expression levels and overall survival (OS) in patients with LGG and THYM (Figure 4E-F). Disease-free survival (DFS) evaluation demonstrated that elevated GPR141 expression served as a favorable prognostic indicator for ACC, UCEC and SKCM (Supplementary Figure 2A-C). Conversely, the expression of GPR141 was associated with poor clinical outcomes in GBM and LGG patients (Supplementary Figure 2D-E).

### The genetic alteration landscape of GPR141 in human pan-cancer

**Figure 5:**
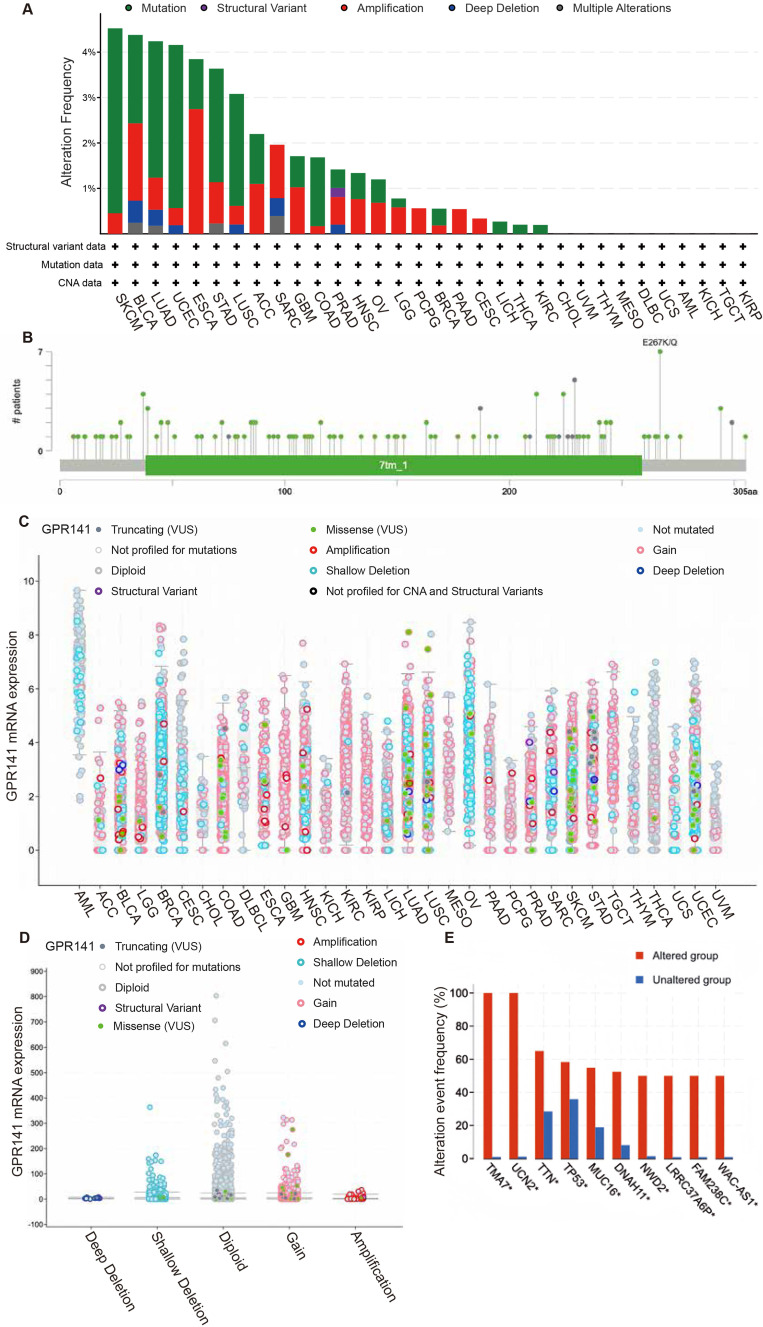
** The genetic alterations of GPR141 in human pan-cancer.** (A) The summary analysis of GPR141 genetic alteration in TCGA PanCancer Atlas Studies. (B) Mutation types, number and sites of GPR141 across protein domains. (C, D) Correlation between the putative CNA of GPR141 and its expression in cancers. (E) The related genes alteration frequency in groups with or without GPR141 alteration.

For comprehensive analysis of GPR141 genomic alterations across various malignancies, we conducted a systematic investigation utilizing the cBioPortal bioinformatics resource, which integrates and processes cancer genomics data from The Cancer Genome Atlas (TCGA) database. The highest frequency of GPR141 alteration showed in SKCM, BLCA, LUAD, UCEC, ESCA, STAD and LUSC (Figure 5A). Genomic analysis revealed that mutational events represented the predominant form of genetic alterations observed in TCGA tumor specimens. Our investigation identified 129 distinct genetic variations in the GPR141 gene, comprising 111 amino acid substitutions, 16 protein-truncating alterations, and 2 gene fusion events (Figure 5B). Notably, the E267K/Q substitution was identified in multiple cancer types, specifically in one BLCA case, two UCEC cases, and four SKCM cases (Figure 5B). So, we thought the E267 is the most frequently altered site within the GPR141 protein structure. Subsequent analysis demonstrated the association between GPR141 copy number alterations (CNAs) and its transcriptional activity across various malignancies (Figure 5C-D). Comparative genomic profiling revealed significant differences in the mutation patterns of several genes, including TMA7, UCN2, TTN, TP53, MUC16, DNAH11, NWD2, LRRC337A6P, FAM238C, and WAC-AS1, between samples with and without GPR141 genomic alterations (Figure 5E).

### Functional enrichment analysis of GPR141 in human pan-cancer

**Figure 6:**
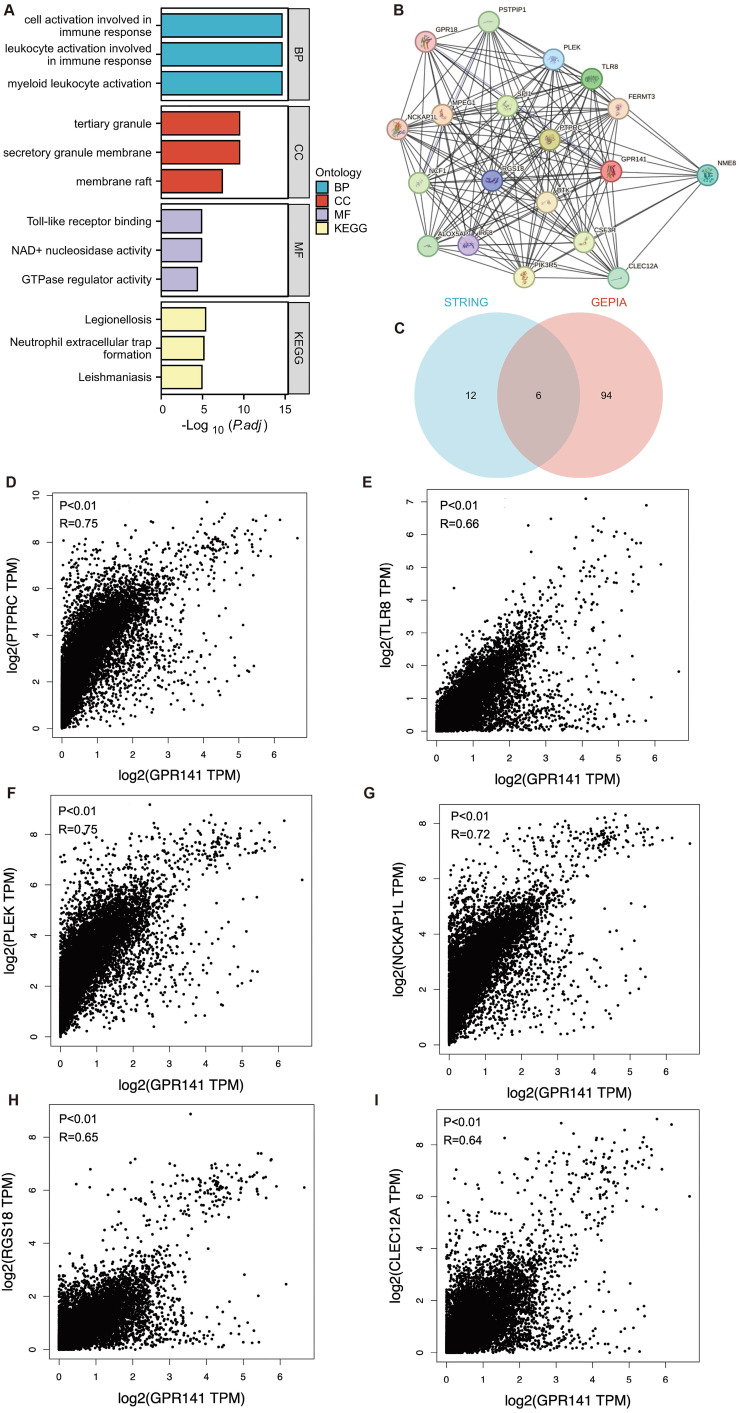
** GPR141-related genes functional enrichment analysis in human pan-cancer.** (A) Gene Ontology (GO) and KEGG analysis of the top 100 genes co-expressed with GPR141 obtained by the GEPIA 2.0. (B) Co-expression network of 18 genes co-expressed with GPR141 obtained by the STRING tool. (C) Venn diagram analysis of genes co-expressed with GPR141 obtained by GEPIA 2.0 and STRING. (D-I) Correlation analysis between GPR141 and PTPRC, TLR8, PLEK, NCKAP1L, RGS18 and CLEC12A conducted by GEPIA 2.0 across all tumor samples from TCGA.

To elucidate the biological functions of GPR141 in cancer development, we employed GEPIA2.0 to identify 100 genes demonstrating the highest co-expression correlation with GPR141 across TCGA malignancies. Functional enrichment analysis, incorporating both Gene Ontology and KEGG pathway assessments, revealed significant associations with immunological activation processes (Figure 6A). Furthermore, protein-protein interaction network analysis through STRING identified 18 functionally associated genes that exhibited co-expression relationships with GPR141 (Figure 6B). We performed Venn diagram analysis of two genes co-expressed with GPR141, and finally obtained six duplicate genes (Figure 6C). We performed correlation analysis of these six genes (PTPRC, TLR8, PLEK, NCKAP1L, RGS18 and CLEC12A) with GPR141 in pan-cancer. The results showed that PTPRC, TLR8, PLEK, NCKAP1L, RGS18 and CLEC12A displayed strong correlation with GPR141 in most cancer types (Figure 6D-I).

### Immunogenomic analysis of GPR141 in human pan-cancer

**Figure 7:**
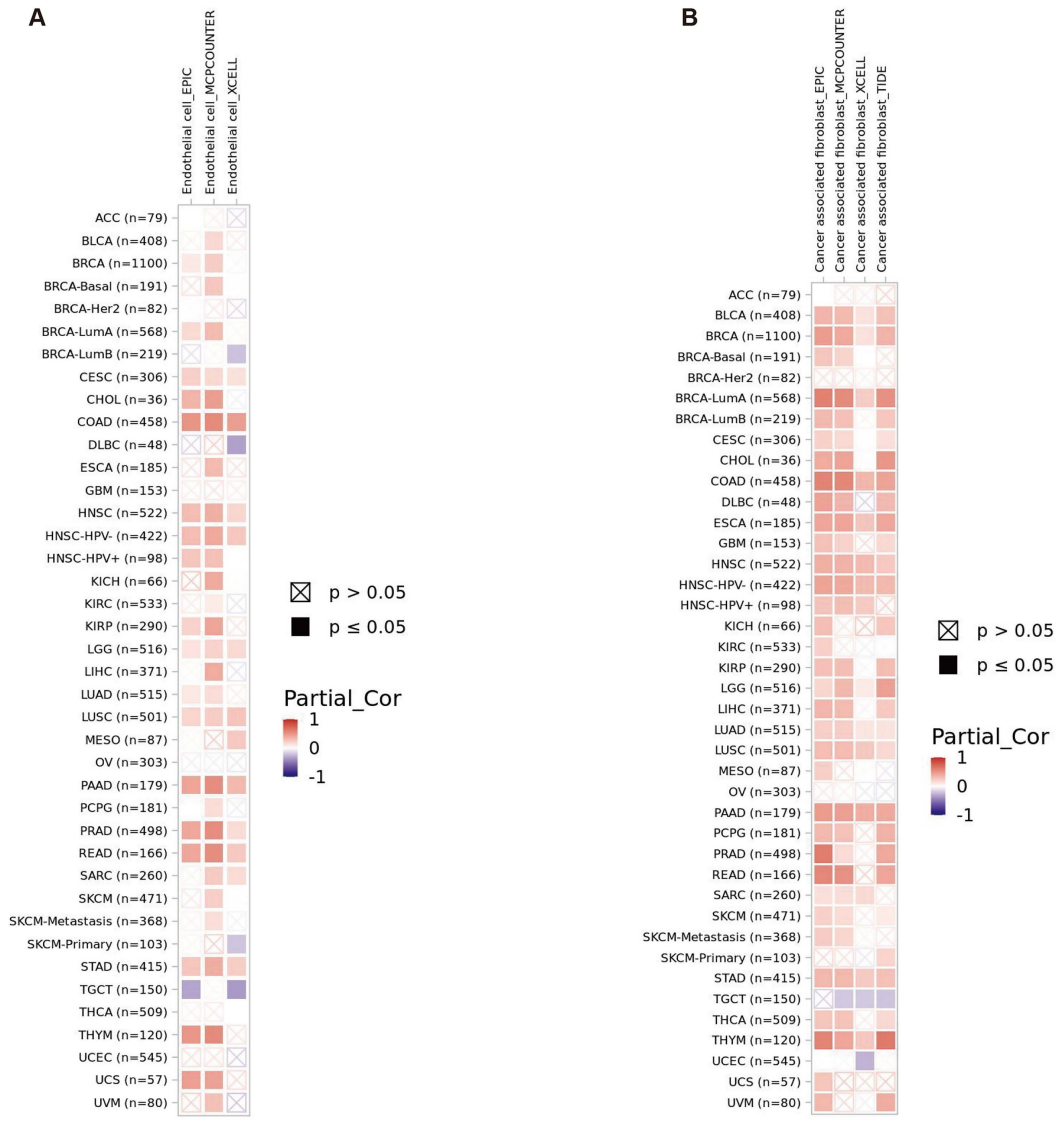
** Correlation between GPR141 expression and immune infiltrates in pan-cancer.** (A, B) The relationship between GPR141 expression level and infiltration of cancer-associated fibroblasts (A) and endothelial cells (B) across all TCGA tumors. The red square represented positive correlation (0-1), while blue square indicated negative correlation (- 1 -0). p value < 0.05 was considered as statistically significant. A cross mean non-significant correlation. The relationship between GPR141 expression and immune infiltration levels across all TCGA cancers analyzed via TIMER2.0 tool.

The gene enrichment analysis showed that GPR141 had a certain relationship with immunity. Given the pivotal significance of immune cell infiltration and immunomodulatory mechanisms in tumorigenesis, we employed the TIMER2.0 analytical platform to investigate potential associations between GPR141 expression patterns and the infiltration density of diverse immune cell populations and endothelial cells across multiple cancer types within the TCGA database. This comprehensive pan-cancer analysis aimed to elucidate the potential immunoregulatory role of GPR141 in the tumor microenvironment. The results showed that GPR141 expression was positively correlated with the infiltration of cancer-associated fibroblasts in BLCA, BRCA (BRCA LumA), COAD, ESCA, HNC-HPV (-), LUAD, LUSC, PAAD, STAD and THYM (Figure 7A). In addition, our investigation identified significant associations between elevated GPR141 expression levels and increased endothelial cell infiltration across several cancer types, including COAD, HNSC-HPV (-), LGG, LUSC, PAAD, PRAD, READ, and STAD (Figure 7B).

Furthermore, our analysis demonstrated significant associations between GPR141 expression patterns and various immunomodulatory molecules, encompassing both immune checkpoint inhibitors and activators, across multiple malignancies. Notably, these correlations were consistently observed in all examined cancer types with the exception of CHOL, COAD, KIRP, LGG, LIHC, PCPG, PRAD and READ (Supplementary Figure 3A-B). Our analysis revealed significant associations between GPR141 expression and immune-related molecules across various malignancies. Regarding major histocompatibility complex (MHC) molecules, the expression profile of GPR141 demonstrated predominantly positive correlations with most MHC components across multiple cancer types, with only a minority of MHC molecules exhibiting a negative correlation (Supplementary Figure 3C). Furthermore, comprehensive pan-cancer analysis indicated that GPR141 expression exhibited substantial associations with numerous chemokine family members, excluding CCL1, CCL16, and CCL27 (Supplementary Figure 3D). Additionally, our investigation demonstrated that the majority of chemokine receptors displayed positive regulatory relationships with GPR141 expression patterns in most analyzed tumor types.

### GPR141 functions as an oncogenic factor in lung cancer

Through reviewing GPR141-related publications and searching the Human Protein Atlas (HPA) database, we identified a lack of reported immunohistochemical evidence regarding GPR141 expression in clinical tissue specimens. To address this knowledge gap, we performed immunohistochemical analysis using clinically obtained lung adenocarcinoma (LUAD) and hepatocellular carcinoma (HCC) samples to comparatively evaluate GPR141 expression patterns between normal and tumor tissues.

**Figure 8:**
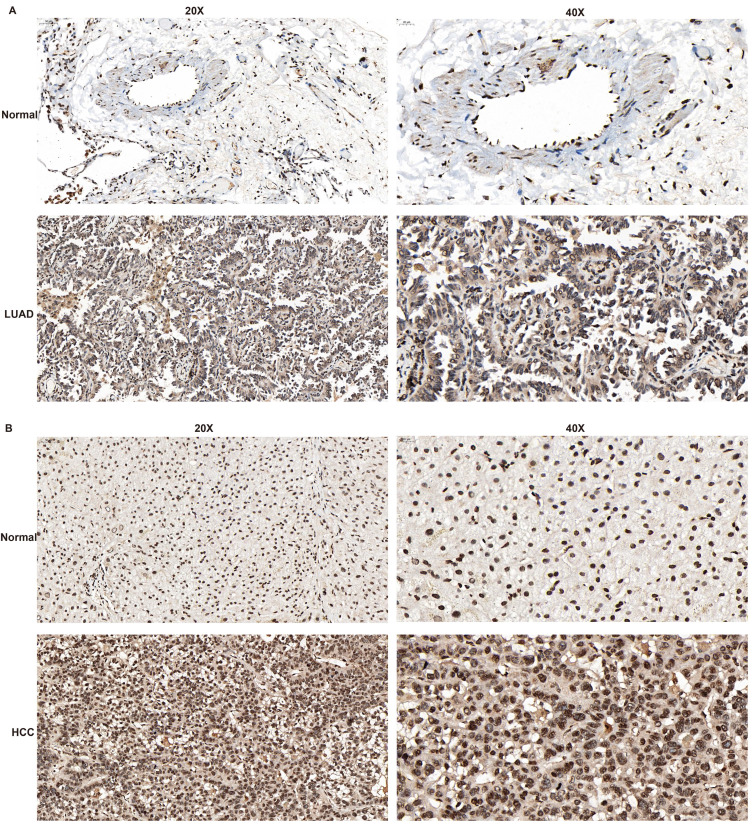
** The expression level of GPR141 in LUAD and HCC tissues.** (A) LUAD. (B) HCC.

Immunohistochemical analysis demonstrated significantly elevated GPR141 expression in both lung adenocarcinoma and hepatocellular carcinoma tissues compared with their normal counterparts, which aligned with our preliminary bioinformatic predictions (Figure 8A-B and Supplementary Figure 4A). We observed that in both lung adenocarcinoma (LUAD) and hepatocellular carcinoma (HCC), GPR141 exhibits predominantly cytoplasmic localization.

**Figure 9:**
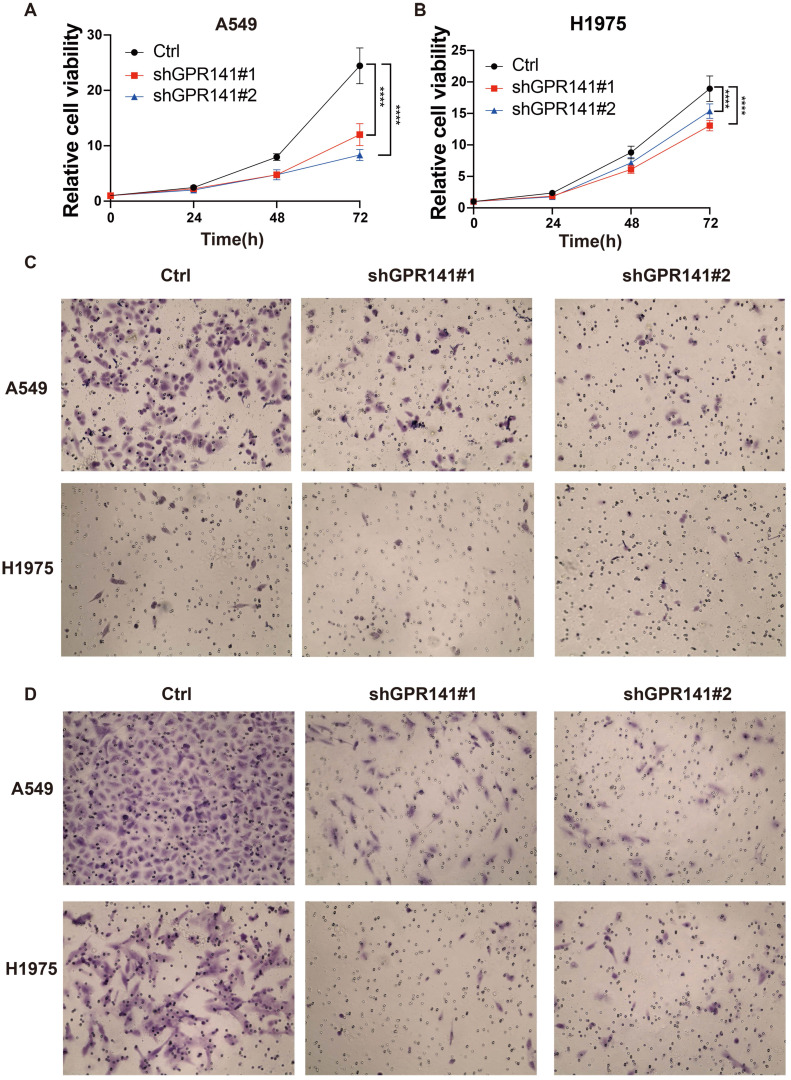
** GPR141 functions as an oncogene in lung adenocarcinoma.** (A) The cell viability in A549 cells stably knocked down of GPR141. (B) The cell viability in H1975 cells stably knocked down of GPR141. (C) The cell migration ability in A549 and H1975 cells stably knocked down of GPR141. (D) The cell invasion ability in A549 and H1975 cells stably knocked down of GPR141.

We detected the protein expression of GPR141 in normal lung epithelial cells and lung adenocarcinoma cells. And the result showed that the expression of GPR141 in lung adenocarcinoma cells was higher than that in normal lung epithelial cells (Supplementary Figure 4B). To explore the physiological role of GPR141 in cancer, we established stable GPR141 knockdown in lung adenocarcinoma cell lines A549 and H1975 (Supplementary Figure 4C-D). We found that knockdown of GPR141 could decreased the cell growth (Figure 9A-B). Moreover, we also found that knockdown of GPR141 could suppressed cell migration and invasion (Figure 9C-D and Supplementary Figure 4E-F). These results demonstrate that GPR141 acts as a tumor-promoting molecule in lung adenocarcinoma by enhancing cancer cell proliferation, migration and invasive capacity.

## Discussion

GPR141 located at chromosome 7p14.1, GPR141 is a seven transmembrane orphan receptor, the ligand of GPR141 is not clear. Recent studies have shown that two articles concerning the association between GPR141 and cancer in PubMed.

The first article reported the discovery of seven novel members of the human G protein-coupled receptor (GPCR) superfamily, designated GPR100, GPR119, GPR120, GPR135, GPR136, GPR141, and GPR142, identified through searches of the human genome database. Phylogenetic analysis indicated these represent additional members of the rhodopsin-type GPCR family. These orphan receptors are predominantly highly conserved across several vertebrate species and exist as single-copy genes. Analysis of expressed sequence tags (ESTs) revealed distinct expression patterns. And the article found that several ESTs for GPR141 were identified in bone marrow and cancer cells, while the expression profiles of other receptors appeared more restricted 37.

The second article published in 2023, utilized breast cancer as a model. Through both *in vivo* and *in vitro* experiments, this study demonstrated that upregulated expression of GPR141 enhances the migratory behavior of breast cancer cells. Furthermore, GPR141 drives oncogenesis *in vitro* and *in vivo* by activating the epithelial-to-mesenchymal transition (EMT), regulating oncogenic mediators, and modulating the p-mTOR/p53 signaling axis 38. At the same time, in multiple sclerosis, when GPR141 is missing, it will damage the immune regulation pathway, which in turn increases the severity of multiple sclerosis 33. These results indicated that GPR141 plays an important role in tumor progression and immune response regulation. However, the potential role of GPR141 in cancers beyond breast cancer remains unvalidated, and no study has yet predicted or analyzed the function of GPR141 across pan-cancer contexts. In this study, we used the data of TCGA database and a variety of bioinformatic websites to analyze and predict the expression and related functions of GPR141 in pan-cancer 39-47.

First, we found that GPR141 is widely expressed in a variety of tissues and differentially expressed in most tumors. At the same time, our study found that GPR141 was associated with the staging of patients' diseases in BLCA, COAD, KICH, SKCM and STAD. To elucidate the clinical significance of GPR141, we conducted an investigation into the association between GPR141 expression levels and patient outcomes. Furthermore, we identified significant correlations between GPR141 expression levels and specific molecular/immune subtypes across cancers, suggesting that targeting these distinct subtypes could provide deeper insights into GPR141's functional roles in tumor biology.

Next, we found that high expression of GPR141 was associated with longer overall survival in HNSC, MESO, SARC and SKCM cancers. In LGG and THYM tumors, the high expression of GPR141 was positively correlated with the shorter overall survival time of patients. We further analyzed the relationship between GPR141 expression level and DFS of patients. The results showed that high expression of GPR141 was associated with longer DFS of ACC and UCEC. And the patients with high expression of GPR141 had shorter DFS in GBM, LGG and SKCM. In this study, we revealed that mutations of GPR141 were most common in SKCM, BLCA, LUAD, UCEC, ESCA, STAD and LUSC. Our genomic analysis revealed that somatic variations and mutational events in the GPR141 gene are prevalent across multiple malignancies. And the frequency of MA7, UCN2, TTN. TP53, MUC16, DNAH11, NWD2, LRRC337A6P, FAM238C, WAC-AS1 alterations was obviously higher in GPR141 alteration group.

Through comprehensive analysis utilizing the GEPIA2.0, we systematically identified multiple genes exhibiting co-expression patterns with GPR141 across diverse tumor types and normal tissues. Functional annotation and pathway enrichment analysis demonstrated that these co-expressed genes were significantly enriched in biological processes related to cellular activation within immune response pathways. We obtained some genes associated with GPR141 through STRING, and analyzed them with the genes we previously obtained from GEPIA. Finally, we found six duplicate genes (PTPRC, TLRB, PLEK, NCKAP1L, PGS18 and CLEC12A). The correlation analysis by GEPIA showed that the six genes were positively correlated with GPR141. These findings paved the way for further exploration of GPR141 molecular function.

In recent years, more and more research has found that there is a close relationship between immunity and tumor, so more and more projects take the research and treatment of tumors on immunity. Our genes enrichment pathways for GPR141-related genes and related literature have suggested that there is a correlation between GPR141 and immune regulation. In the present investigation, we systematically evaluated the immunomodulatory role of GPR141 through comprehensive analysis of tumor microenvironment characteristics. Our findings demonstrated statistically significant associations between GPR141 expression levels and immune cell infiltration patterns, particularly involving cancer-associated fibroblasts (CAFs) and endothelial cells (ECs) in specific tumor types. Furthermore, our analysis revealed substantial regulatory relationships between GPR141 and multiple genes involved in immunomodulatory pathways across various malignancies. These collective results indicate that GPR141 may serve as a promising therapeutic target for immunotherapeutic interventions, potentially improving clinical outcomes in cancer patients. However, the mechanism interaction between GPR141 expression and immune checkpoint regulation needs further experimental exploration.

Finally, the immunohistochemical findings conclusively confirmed elevated GPR141 expression in clinical specimens of lung adenocarcinoma (LUAD) and hepatocellular carcinoma (HCC) compared to adjacent normal tissues. To delineate the biological functions of GPR141, we established stable GPR141-knockdown cell lines in two LUAD cell models (A549 and H1975). Functional characterization revealed that GPR141 knockdown significantly suppressed tumor cell proliferation, migration, and invasive capacities. These results established the role of GPR141 as a carcinogenic driver in the pathogenesis of LUAD.

In conclusion, our comprehensive investigation employed multiple bioinformatics approaches to systematically evaluate the expression profiles, prognostic significance, mutational landscape, immunoregulatory functions, and associated molecular pathways of GPR141 across various malignancies. The findings demonstrate that GPR141 exhibits considerable diagnostic potential across different cancer types. Furthermore, our data suggest that GPR141 may serve as a promising candidate for both prognostic prediction and immune-related biomarker development in oncology. Through multidimensional analysis, this research provides novel insights into the multifaceted roles of GPR141 in cancer biology, offering a more comprehensive understanding of its involvement in tumor development and progression.

## Supplementary Material

Supplementary figures and tables.

## Abbreviations

ACC: Adrenocortical carcinoma
ALL: Acute Lymphoblastic Leukemia
BLCA: Bladder Urothelial Carcinoma
BRCA: Breast invasive carcinoma
CESC: Cervical squamous cell carcinoma and endocervical adenocarcinoma
CHOL: Cholangiocarcinoma
COAD: Colon adenocarcinoma
COADREAD/CRC: Colorectal cancer
DLBC: Lymphoid Neoplasm Diffuse Large B-cell Lymphoma
ESCA: Esophageal carcinoma
GBM: Glioblastoma multiforme
HNSC: Head and Neck squamous cell carcinoma
KICH: Kidney Chromophobe
KIRC: Kidney renal clear cell carcinoma
KIRP: Kidney renal papillary cell carcinoma
LAML: Acute Myeloid Leukemia
LGG: Brain Lower Grade Glioma
LIHC: Liver hepatocellular carcinoma
LUAD: Lung adenocarcinoma
LUSC: Lung squamous cell carcinoma
MESO: Mesothelioma
OV: Ovarian serous cystadenocarcinoma
PAAD: Pancreatic adenocarcinoma
PCPG: Pheochromocytoma and Paraganglioma
PRAD/PC: Prostate adenocarcinoma
RB: Retinoblestoma
RCC: Renal cell carcinoma
READ: Rectum adenocarcinoma
SARC: Sarcoma
SKCM: Skin Cutaneous Melanoma
STAD: Stomach adenocarcinoma
TGCT: Testicular Germ Cell Tumors
THCA: Thyroid carcinoma
THYM: Thymoma
UCEC: Uterine Corpus Endometrial Carcinoma
UCS: Uterine Carcinosarcoma
UVM/UM: Uveal Melanoma
