# Supplementary material for: Pan-Cancer Analysis of GPR141: Unveiling its prognostic significance, immune microenvironment interactions, and therapeutic potential
Source: J Cancer. 2025 Aug 11;16(12):3712–28. doi: 10.7150/jca.118222 (PMC12435298; doi:10.7150/jca.118222)
Supplement: Supplementary file 1 — Supplementary figures and tables. [file jcav16p3712s1.pdf]

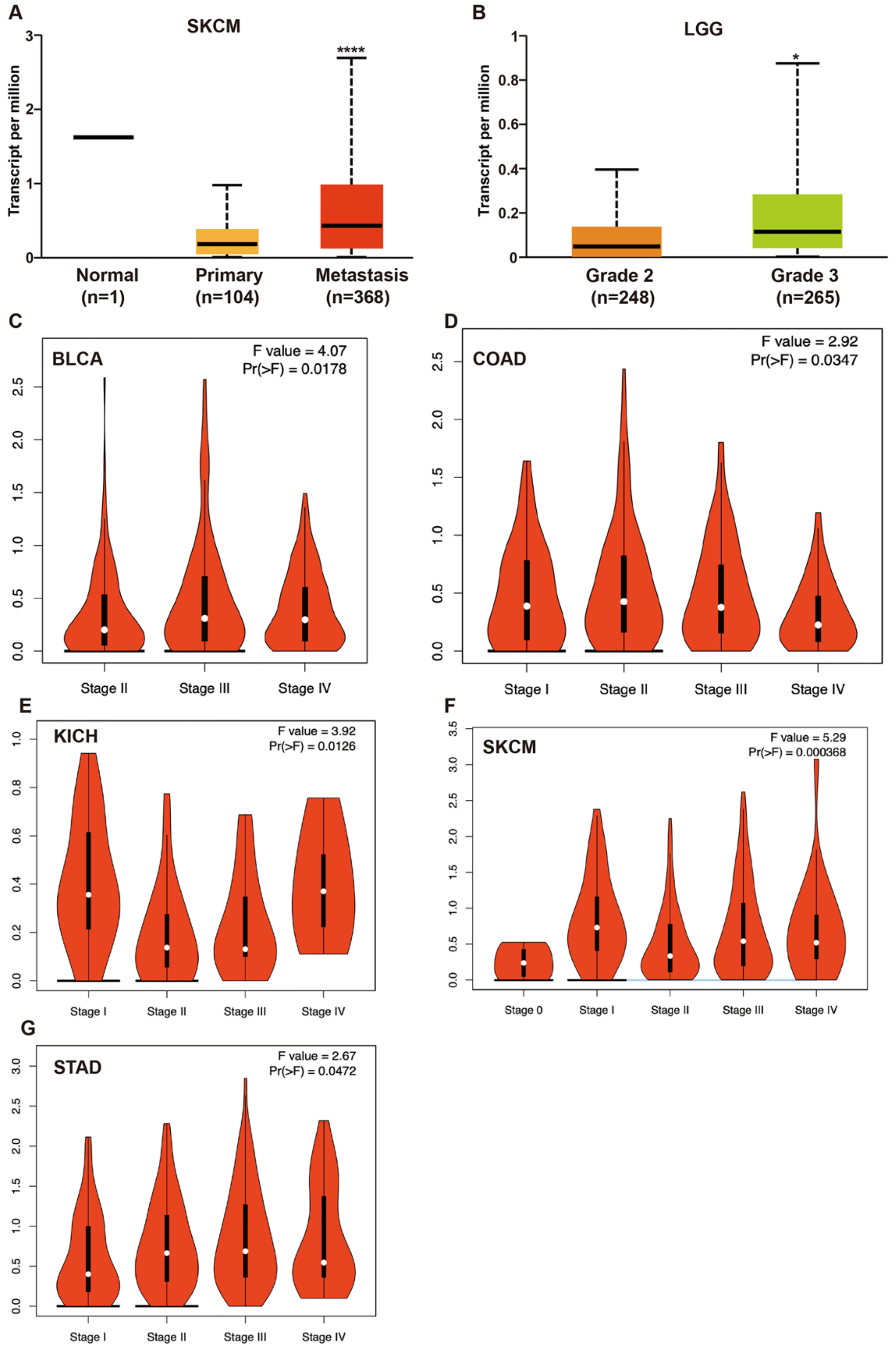

Supplementary Figure 1. The mRNA expression levels of GPR141 in human pan-cancer.

(A) GPR141 mRNA expression level in primary and metastasis of SKCM. (B) GPR141 mRNA expression level in Grade 2 and Grade 3 of LGG. (C-G) Analysis of GPR141 mRNA expression level by the main pathological stages using GEPIA2.0.

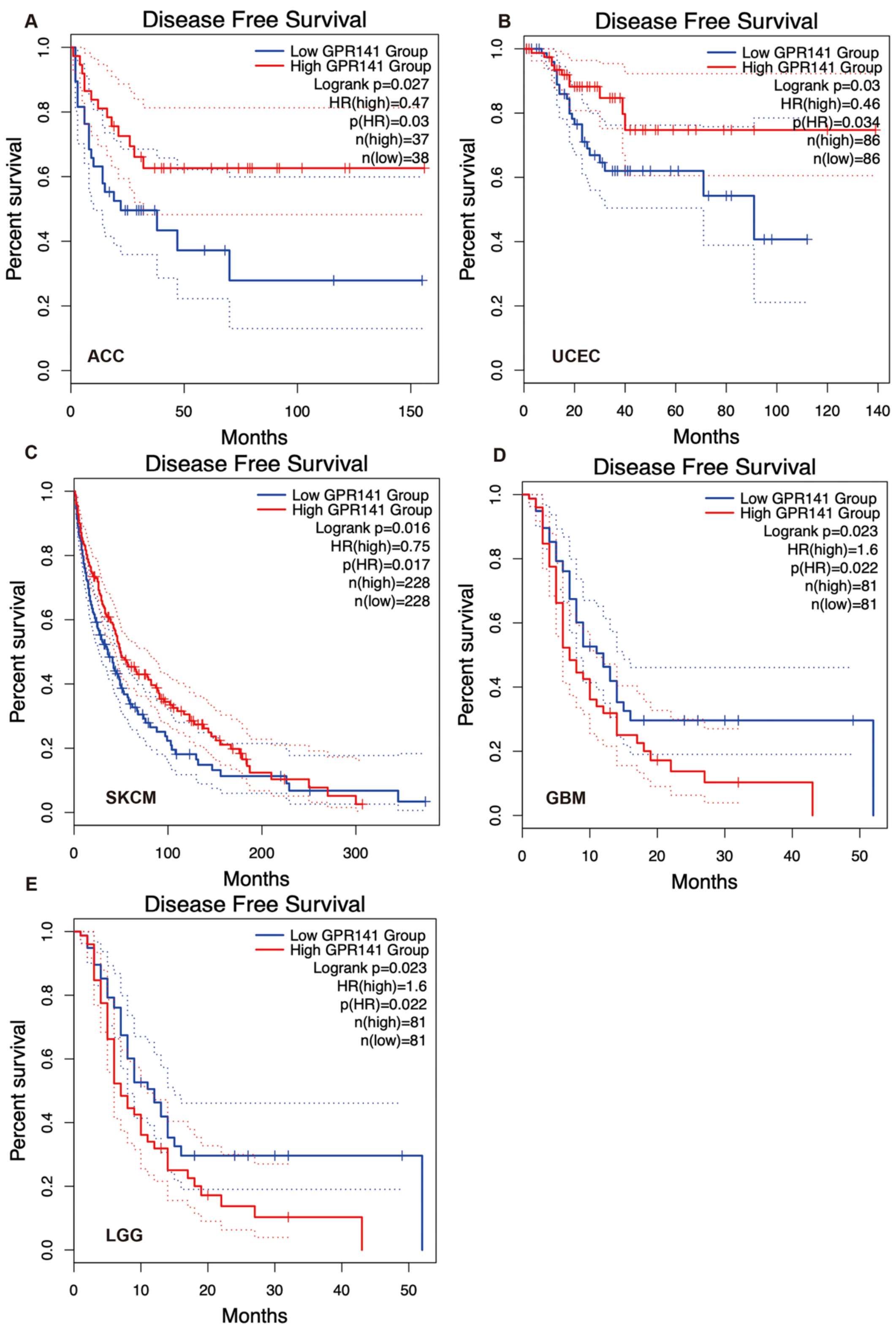

Supplementary Figure 2. Correlation between GPR141 expression and disease-free survival in human pan-cancer.

Kaplan–Meier curves for patient's disease-free survival classified by different expression level of GPR141 in ACC(A), UCEC(B), SKCM(C), GBM(D) and LGG(E).

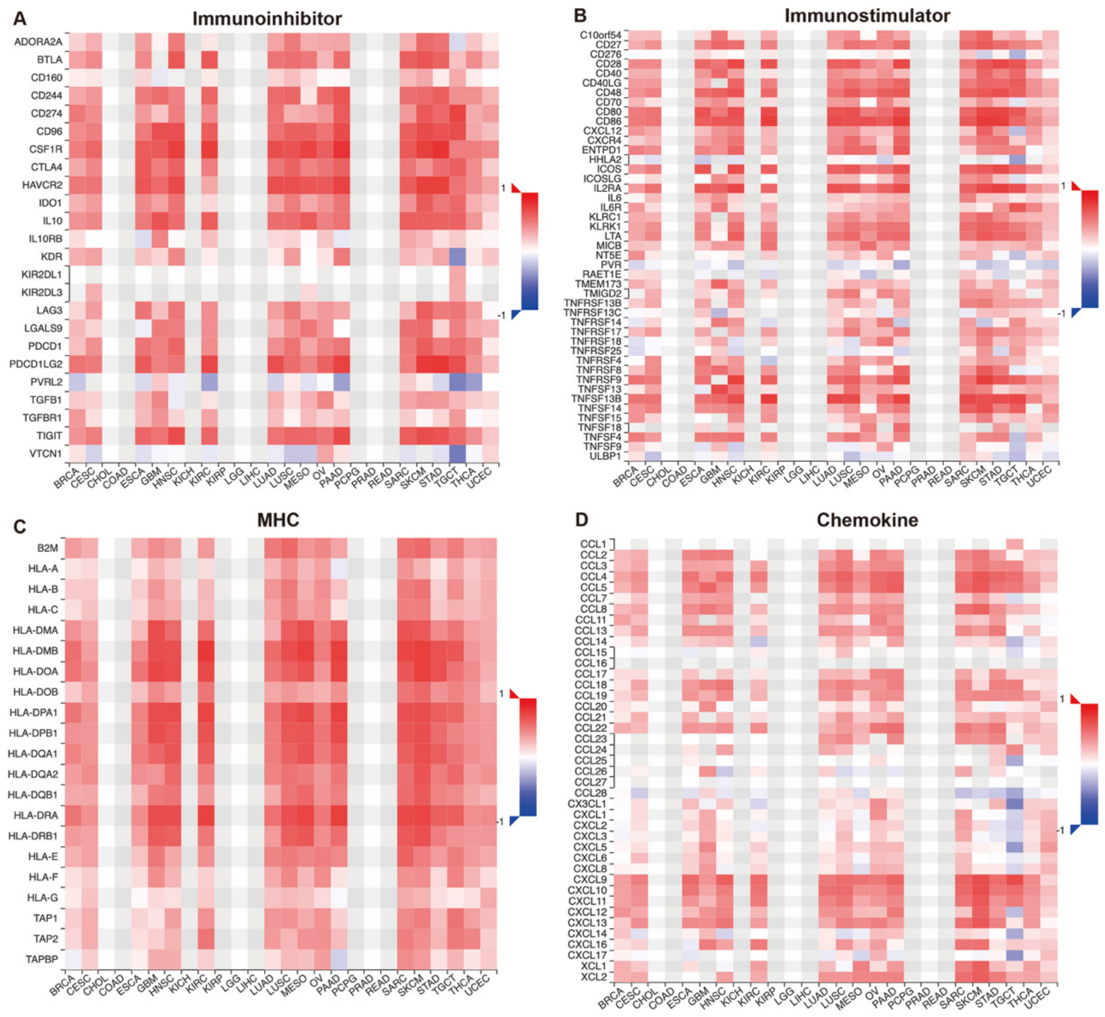

Supplementary Figure 3. The correlation between GPR141 and immunoregulation-related genes in human pan-cancer.

Correlations between GPR141 expression and immunos timulators (A) immunoinhibitors (B) MHC molecules (C) chemokines (D) and chemokine receptors (E) were obtained via the TISIDB database.

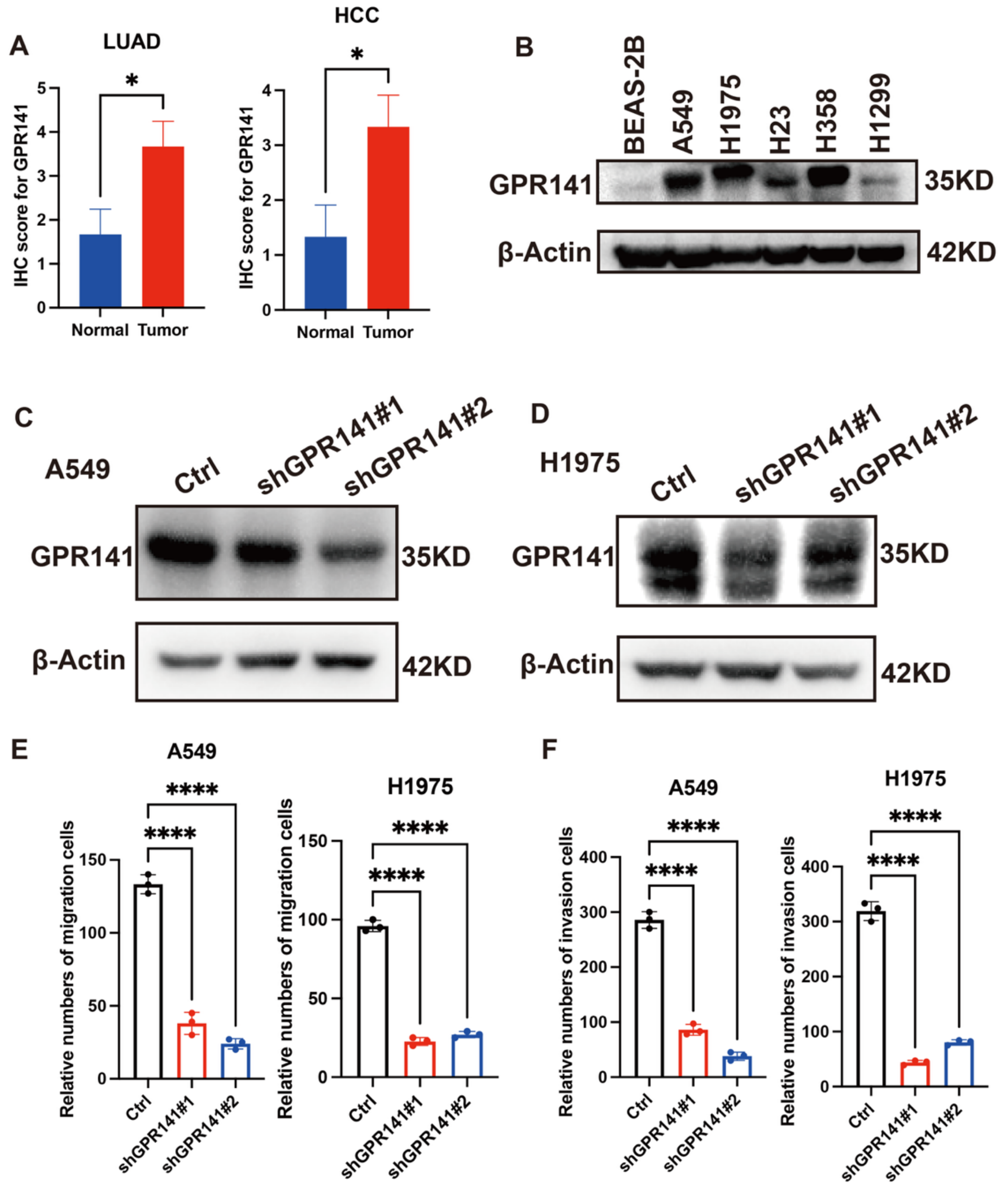

Supplementary Figure 4. Western Blot Analysis and Transwell Migration/Invasion Assay Quantification.

(A) The IHC score for GPR141 in LUAD and LCC. (B) We collected the protein of the normal lung epithelial cell line BEAS-2B and the lung adenocarcinoma cell lines A549, H1975, H23, H358, and H1299. GPR141 expression levels were assessed by western blot analysis. (C)The expression of GPR141 knocked down in lung adenocarcinoma cells A549 by western blot. (D) The expression of GPR141 knocked down in lung adenocarcinoma cells H1975 by western blot. (E) Quantitative analysis of migration ability in A549 and H1975 cells stably knocked down of GPR141. (F) Quantitative analysis of invasion ability in A549 and H1975 cells stably knocked down of GPR141.

Supplementary Table 1. The abbreviation of 37 cancer types analyzed in this study.

Abbreviation	Cancer types
ACC	Adrenocortical carcinoma
ALL	Acute Lymphoblastic Leukemia
BLCA	Bladder Urothelial Carcinoma
BRCA	Breast invasive carcinoma
CESC	Cervical squamous cell carcinoma and endocervical adenocarcinoma
CHOL	Cholangiocarcinoma
COAD	Colon adenocarcinoma
COADREAD/CRC	Colorectal cancer
DLBC	Lymphoid Neoplasm Diffuse Large B-cell Lymphoma
ESCA	Esophageal carcinoma
GBM	Glioblastoma multiforme
HNSC	Head and Neck squamous cell carcinoma
KICH	Kidney Chromophobe
KIRC	Kidney renal clear cell carcinoma
KIRP	Kidney renal papillary cell carcinoma
LAML	Acute Myeloid Leukemia
LGG	Brain Lower Grade Glioma
LIHC	Liver hepatocellular carcinoma
LUAD	Lung adenocarcinoma
LUSC	Lung squamous cell carcinoma
MESO	Mesothelioma
OV	Ovarian serous cystadenocarcinoma
PAAD	Pancreatic adenocarcinoma
PCPG	Pheochromocytoma and Paraganglioma
PRAD/PC	Prostate adenocarcinoma
RB	Retinoblestoma
RCC	Renal cell carcinoma
READ	Rectum adenocarcinoma
SARC	Sarcoma
SKCM	Skin Cutaneous Melanoma
STAD	Stomach adenocarcinoma
TGCT	Testicular Germ Cell Tumors
THCA	Thyroid carcinoma
THYM	Thymoma
UCEC	Uterine Corpus Endometrial Carcinoma
UCS	Uterine Carcinosarcoma
UVM/UM	Uveal Melanoma
